# Alcohol, smoking, recreational drug use and association with virological outcomes among people living with HIV: cross‐sectional and longitudinal analyses

**DOI:** 10.1111/hiv.13156

**Published:** 2021-10-11

**Authors:** Timothy P. W. Jones, Fiona C. Lampe, Alejandro Arenas‐Pinto, Colette Smith, Jeff McDonnell, Lewis Haddow, Margaret Johnson, Elaney Yousef, Monica Lascar, Anna Maria Geretti, Lorraine Sherr, Simon Collins, Andrew N. Phillips, Alison J. Rodger, the ASTRA Study Group

**Affiliations:** ^1^ Royal Free Hospital Foundation Trust London UK; ^2^ Research Department of Infection & Population University College London London UK; ^3^ Brighton and Sussex University Hospitals Brighton UK; ^4^ Barts Health NHS Trust London UK; ^5^ Institute of Infection and Global Health University of Liverpool Liverpool UK; ^6^ HIV i‐Base London UK

**Keywords:** adherence, alcohol dependence, viral non‐suppression, recreational drugs, smoking

## Abstract

**Objectives:**

There is increasing evidence to suggest that people living with HIV (PLWH) have significant morbidity from alcohol, recreational drug use and cigarette smoking. Our aim was to report associations of these factors with antiretroviral therapy (ART) non‐adherence, viral non‐suppression and subsequent viral rebound in PLWH.

**Methods:**

The Antiretroviral Sexual Transmission Risk and Attitudes (ASTRA) study recruited PLWH attending eight outpatient clinics in England between February 2011 and December 2012. Data included self‐reported excessive drinking (estimated consumption of > 20 units of alcohol/week), alcohol dependency (CAGE score ≥ 2 with current alcohol consumption), recreational drug use (including injection drug use in the past 3 months), and smoking status. Among participants established on ART, cross‐sectional associations with ART non‐adherence [missing ≥2 consecutive days of ART on ≥2 occasions in the past three months] and viral‐non suppression [viral load (VL) > 50 copies/mL] were assessed using logistic regression. In participants from one centre, longitudinal associations with subsequent viral rebound (first VL > 200 copies/mL) in those on ART with VL ≤ 50 copies/mL at baseline were assessed using Cox regression during a 7‐year follow‐up.

**Results:**

Among 3258 PLWH, 2248 (69.0%) were men who have sex with men, 373 (11.4%) were heterosexual men, and 637 (19.6%) were women. A CAGE score ≥ 2 was found in 568 (17.6%) participants, 325 (10.1%) drank > 20 units/week, 1011 (31.5%) currently smoked, 1242 (38.1%) used recreational drugs and 74 (2.3%) reported injection drug use. In each case, prevalence was much more common among men than among women. Among 2459 people on ART who started at least 6 months previously, a CAGE score ≥ 2, drinking > 20 units per week, current smoking, injection and non‐injection drug use were all associated with ART non‐adherence. After adjusting for demographic and socioeconomic factors, CAGE score ≥ 2 [adjusted odds ratio (aOR) = 1.52, 95% confidence interval (CI): 1.09–2.13], current smoking (aOR = 1.58, 95% CI: 1.10–2.17) and injection drug use (aOR = 2.11, 95% CI: 1.00–4.47) were associated with viral non‐suppression. During follow‐up of a subset of 592 people virally suppressed at recruitment, a CAGE score ≥ 2 [adjusted hazard ratio (aHR) = 1.66, 95% CI: 1.03–2.74], use of 3 or more non‐injection drugs (aHR = 1.82, 95% CI: 1.12–3.57) and injection drug use (aHR = 2.73, 95% CI: 1.08–6.89) were associated with viral rebound.

**Conclusions:**

Screening and treatment for alcohol, cigarette and drug use should be integrated into HIV outpatient clinics, while clinicians should be alert to the potential for poorer virological outcomes.

## INTRODUCTION

Effective antiretroviral therapy (ART) and increased HIV testing have dramatically increased survival in people living with HIV (PLWH). However, lifestyle factors such as alcohol consumption, smoking and recreational drug use may have an impact on adherence to ART and HIV outcomes as well as increasing risk of non‐HIV‐related morbidity. Previous questionnaire studies have suggested that alcohol, smoking and recreational drug use are prevalent in PLWH [1, 2, 3, 4, 5, 6, 7].

The WHO attribute 5.9% of global deaths to alcohol consumption [8]. There is evidence that alcohol intake may have a greater impact on the mortality and morbidity of PLWH compared with the general population, although the causes for this remain unclear [9]. While there is evidence that higher alcohol consumption is associated with ART non‐adherence among PLWH [1], the impact of alcohol on virological outcomes of ART *per se* is less clear. Although several studies reported a positive association between high alcohol intake and viral load rebound [2, 5, 10, 11, 12, 13], others have not found significant associations [1, 14, 15]. Lack of consistent definitions of what constitutes hazardous or harmful alcohol use, the potential for non‐linear associations with amount of alcohol and, in some cases, small sample sizes have hampered interpretation of this literature.

Reduced adherence to ART and higher viral loads have likewise been associated with cigarette smoking [16] and use of recreational drugs, including ‘chemsex’ [4]. There is evidence that PLWH who smoke are particularly susceptible to lung damage from smoking, with those stable on treatment expected (based on modelling analysis) to lose more years to smoking than to HIV [17]. Although people who inject drugs are known to have poor health outcomes, the effects of non‐injected recreational drug use in PLWH is less clear, although recent studies suggest that this may also have a negative impact on adherence [3, 18]. In combination, these findings may indicate that adverse lifestyle factors have significant implications for increased morbidity in PLWH, as well as potential implications for HIV treatment outcomes and onward transmission in PLWH on ART.

Much of the literature has focused on PLWH already identified as high risk for alcohol and drug use disorders [19]. Furthermore, few studies have reported on the use of drugs, alcohol, smoking and the association with virological outcomes among PLWH in the UK. Different societal views and practices make unselected country‐specific data invaluable in measuring the impact of such lifestyle factors and in guiding the public health response. The aim of this study was to use data from the Antiretroviral Sexual Transmission Risk and Attitudes (ASTRA) study [20] to report levels of alcohol, smoking and recreational drug use among PLWH in England, to assess associations with demographic socioeconomic, health and HIV‐related factors, and to examine the associations with ART non‐adherence, viral non‐suppression and subsequent virological rebound over a 7‐year period.

## METHODS

The ASTRA study has been described previously [20]. In brief it is a multicentre cross‐sectional, questionnaire study of 3258 HIV‐positive participants aged 18 years and over, attending eight HIV outpatient clinics in England, with a subsequent longitudinal component. The participants were enrolled between 1 February 2011 and 31 December 2012.

Participants self‐completed a paper questionnaire that included items on demographics (including gender, sexual orientation, age, ethnicity, relationship status), socio‐economic factors (education, employment, housing, money for basic needs), health and lifestyle factors (including smoking, alcohol use, recreational drug use, symptoms of depression and anxiety), sexual behaviour (including recent condom‐less sex and number of new partners) and HIV‐related factors (ART treatment status including previous and current ART use, start date and non‐adherence). The most recent plasma HIV‐1 RNA level (viral load, VL) and CD4 cell count were recorded from clinic records for all participants.

In this paper, baseline cross‐sectional analyses are based on the whole ASTRA study sample (*N* = 3258). The longitudinal analysis in this paper uses clinic data of serial viral load measurements from 802 individuals who consented to linkage (of 899 recruited) from one London clinic only (Royal Free Hospital). These follow‐up data cover the period from baseline questionnaire completion until November 2018 for 802 people. Data on smoking, alcohol and recreational drug use from these individuals were obtained only from the original ASTRA questionnaire.

### Lifestyle factors

Two alcohol questionnaires were used in the study. The CAGE questionnaire is a standardized four‐item validated questionnaire that identities alcohol dependency; a score of two or more together with reported current alcohol consumption was used as evidence of dependency [21]. A modified version of AUDIT‐C questionnaire (first two questions only) measured frequency of alcohol intake and number of units typically consumed during a drinking session from which a score was derived (range 0–8) according to standard methods [22]. In addition, a variable approximating to consumption of > 20 units of alcohol/week was defined as five or more units at least four times per week or 10 or more units two to three times per week.

Participants were asked about recreational drug use in the previous 3 months and were asked to specify which drugs had been used from a list of 18, including both official and slang names. They were also asked about injection drug use in the past 3 months [3]. A four‐category drug use variable was derived: no drug use; non‐injection use of one or two drugs; non‐injection use of three or more drugs; and injection drug use.

Participants were asked if they currently or previously smoked cigarettes regularly (at least one/day); smoking status was classified as current, ex or never.

### Other factors

Men were classified as men who have sex with men (MSM) if they self‐identified as gay or bisexual or reported sex with another man in the preceding 3 months. Social support was self‐reported using a modified (five‐question) version of the Duke‐UNC Functional Social Support Questionnaire [23]. The maximum score was 25 and defined as ‘most’ supported; 15–24 was defined as ‘medium’ support, and 5–14 as ‘least’ supported. A Patient Health Questionnaire‐9 (PHQ‐9) total score of 10 or more was used to define depressive symptoms; a score of 10 or more on the General Anxiety Disorder‐7 (GAD‐7) quesitonaire defined anxiety symptoms.

Participants were also asked about sexual practices in the 3 months before study recruitment. The following sexual behaviours were asked about: any anal or vaginal sex, condom‐less sex, lower condom use with casual partners defined as ‘strongly’ or ‘tend to’ agree to the statement ‘I am less likely to use a condom with a casual partner’ (no recall period), and the number of new sexual partners in the previous year (classified as > 10 or ≤ 10). Missing data (< 5% for all variables) were excluded from analysis.

### Cross‐sectional analyses

The distribution of alcohol, smoking and recreational drug use variables is presented, and associations with demographic, socioeconomic, health, HIV‐related and lifestyle factors were assessed using χ^2^ tests and unadjusted odds ratios.

The unadjusted and adjusted associations of the lifestyle factors with non‐adherence to ART were assessed among all participants on ART at the time of the questionnaire who started ART at least 6 months previously. Non‐adherence was defined as missing ≥ 2 consecutive days of ART on two or more occasions in the past 3 months. This definition was chosen to capture significant non‐adherence likely to be clinically important. Associations with HIV viral non‐suppression (defined as VL > 50 copies/mL using the clinic‐documented value) were assessed in those who were on ART at the time of the questionnaire and had started ART at least 6 months prior to the relevant VL measurement. Unadjusted and adjusted associations were assessed using logistic regression analysis. In the multivariable analyses, associations were first adjusted for core demographic factors: gender/sexual orientation, age, and ethnicity before additionally adjusting for education and financial hardship – indicators of socioeconomic status. Results are presented as odds ratios with 95% confidence interval (CI); likelihood ratios were used to calculate statistical significance.

### Longitudinal analysis

In the subset of participants from the Royal Free centre who consented to data linkage were on ART at the time of the questionnaire, had started ART at least 6 months prior to the baseline VL measurement and had a VL ≤ 50 copies/mL at study entry, we assessed the association of alcohol, smoking and recreational drug use with subsequent viral rebound (defined as first VL> 200 copies/mL) during the follow‐up period (until the end of November 2018), using Cox proportional hazards regression. Unadjusted and adjusted analyses were performed, using the same adjustment strategies as for the cross‐sectional analyses. Results are presented as hazard ratios with 95% CIs. Likelihood ratios were used to assess statistical significance.

### Ethics committee approval

The research protocol and all versions of the study documents (information sheet, consent form and questionnaire) were approved by the North West London REC 2 research ethics committee (ref 10/H0720/70). Based on these documents, the study subsequently received permission for clinical research at all participating National Health Service sites.

## RESULTS

Of the 5112 PLWH approached to take part in the study, 3258 were recruited and completed the questionnaire (response rate 64%) of whom 2248 were MSM, 373 were heterosexual men and 637 were women. Participants' characteristics are shown in Table 1.

**TABLE 1:** Participant characteristics and associations with alcohol use and dependency, recreational drug use and cigarette smoking

Demographics	Participant cohort		Alcohol dependence: cage score ≥ 2 and current alcohol consumption					> 20 units alcohol/week from AUDIT					Recreational drug use (in the past 3 months)					Current smoker				
	*n* = 3258	Column %	568/3258 (17.4%)	Column %	OR	95% CI	*P*‐value	325/3258 (10.0%)	Column %	OR	95% CI	*P*‐value	1242/3258 (38.1%)	Column %	OR	95% CI	*P*‐value	1011/3258 (31.0%)	Column %	OR	95% CI	*P*‐value
Male	2621/3258	80.4%	503/568	88.6%	1.45	1.09–1.93	0.0081	314/325	96.6%	7.57	4.10–13.98	< 0.0001	1198/1242	96.5%	11.30	8.13–15.83	< 0.0001	944/1011	93.4%	4.69	3.57–6.16	< 0.0001
Mean age (year) (SD)	45.2	(9.63)	44.3	(8.72)				46.4	(9.45)				43.7	(9.06)				44.0	( 9.07)			
Age > 50 years	971/3258	29.8	136/568	23.9%	0.70	0.57–0.87	0.0009	112/325	34.5%	1.27	1.00–1.63	0.0492	294/1242	23.7%	0.61	0.52–0.72	< 0.0001	253/1011	25.0%	0.71	0.60–0.84	0.0001
Median CD4 lymphocyte count (× 10^9^/mL) (IQR)	537	(390–721)	540	(580–736)				539	(392–729)				556	(418–757)				559	(402–763)			
CD4 < 500 copies/mL	1388/3235	42.9%	323/568	56.9%	1.05	0.87–1.27	0.6023	189/325	58.2%	0.96	0.76–1.21	0.7384	487/1234	38.7%	0.76	0.66–0.88	0.0002	402/1011	40.0%	0.85	0.73–0.99	0.0397
Gender/sexual orientation																						
MSM	2248/3258	69.0%	435/568	76.6%	1		0.0180	282/325	86.8%	1		< 0.0001	1138/1242	91.6%	1		< 0.0001	829/1011	82.0%	1		< 0.0001
Heterosexual men	373/3258	11.4%	68/568	12.0%	1.16	0.87–1.56	—	32/325	9.9%	0.67	0.46–0.98	—	60/1242	4.8%	0.18	0.14–0.25	—	115/1011	11.4%	0.79	0.62–1.00	—
Women	637/3258	19.6%	65/568	11.4%	0.49	0.53–0.94	—	11/325	3.4%	0.12	0.07–0.23	—	44/1242	3.5%	0.07	0.05–0.10	—	67/1011	6.6%	0.21	0.16–0.27	—
Ethnic origin																						
White	2220/3185	69.7%	434/563	77.1%	1		0.1803	291/324	89.8%	1		< 0.0001	1073/1242	86.4%	1		< 0.0001	819/1011	81.0%	1		< 0.0001
Black African	614/3185	19.2%	84/563	14.9%	1.00	0.76–1.30	—	14/324	4.3%	0.16	0.09–0.28	—	21/1242	1.7%	0.04	0.02–0.06	—	55/1011	5.4%	0.17	0.13–0.23	—
Black other	125/3185	3.9%	18/563	3.2%	0.81	0.48–1.35	—	5/324	1.5%	0.29	0.12–0.71	—	45/1242	3.6%	0.60	0.41–0.88	—	48/1011	4.7%	1.13	0.77–1.64	—
All other	226/3185	7.1%	27/563	4.8%	0.65	0.42–0.99	—	14/324	4.3%	0.44	0.25–0.77	—	103/1242	8.3%	0.56	0.44–0.72	—	89/1011	8.8%	0.73	0.36–0.96	—
ART status																						
On ART	2771/3202	86.5%	466/564	82.6%	1		0.0297	269/322	83.5%	1		0.1636	1033/1234	83.7%	1	—	< 0.0001	837/1005	83.3%	1	—	0.0011
Stopped ART	65/3202	2.0%	13/564	2.3%	0.92	0.71–2.56	—	10/322	3.1%	2.26	0.85–3.34	—	20/1234	1.6%	0.75	0.44–1.27	—	23/1005	2.3%	0.75	0.39–2.10	—
Never on ART	366/3202	11.4%	85/564	15.1%	1.43	1.09–1.86	—	43/322	13.3%	1.61	0.89–1.76	—	103/1234	14.7%	1.65	1.32–2.05	—	145/1005	14.4%	1.52	1.21–1.90	—
Social support																						
Most (25)	1042/3258	32.0%	147/559	26.3%	1		0.0002	90/325	27.7%	1		0.0710	385/1236	31.1%	1		0.3891	288/999	28.8%	1		0.0003
Medium (15–24)	1534/3258	47.1%	287/559	51.3%	1.39	1.12–1.73	—	174/325	53.5%	1.36	1.04–1.78	—	591/1236	47.8%	1.07	0.91–1.26	—	485/999	48.5%	1.22	1.02–1.45	—
Least (5–14)	620/3258	19.0%	125/559	22.4%	1.73	1.31–2.26	—	61/325	18.8%	1.15	0.82–1.62	—	260/1236	21.0%	1.23	1.01–1.51	—	226/999	22.6%	1.52	1.22–1.87	—
Mental health symptoms																						
Depression (PHQ‐9 score ≥ 10)	884/3258	27.1%	207/568	36.4%	1.92	1.57–2.35	< 0.0001	101/325	31.1%	1.24	0.96–1.59	0.0930	232/1242	18.7%	1.24	1.07–1.46	0.0060	244/1011	24.1%	1.80	1.53–2.12	< 0.0001
Anxiety (GAD‐7 score ≥ 10)	715/3258	21.9%	172/568	30.3%	1.71	1.39–2.09	< 0.0001	86/325	26.5%	1.32	1.01–1.71	0.0382	272/1242	21.9%	1.00	0.84–1.18	0.9604	290/1011	28.7%	1.75	1.47–2.09	< 0.0001
Money for basic needs																						
Always	1392/3193	43.6%	254/564	45.0%	1		0.0265	165/324	50.9%	1		0.0153	600/1228	48.9%	1		< 0.0001	369/994	37.1%	1		< 0.0001
Sometimes or mostly	1401/3193	43.9%	224/564	39.7%	0.86	0.71–1.05	—	118/324	36.4%	0.69	0.54–0.89	—	502/1228	40.9%	0.74	0.63–0.86	—	474/994	47.7%	1.44	1.23–1.70	—
No	400/3193	12.5%	86/564	15.3%	1.25	1.32–2.35	—	41/324	12.7%	0.87	0.61–1.25	—	126/1228	10.3%	0.61	0.48–0.77	—	151/994	15.2%	1.73	1.37–2.20	—
Education																						
No Qualifications	371/3172	11.7%	68/562	12.1%	1		0.8232	37/322	11.5%	1		0.7791	117/1224	9.6%	1		< 0.0001	133/985	13.5%	1		< 0.0001
Other non‐university qualifications	1484/3172	46.8%	258/562	45.9%	0.87	0.64–1.18	—	145/322	45.0%	0.95	0.65–1.39	—	589/1224	48.1%	1.43	1.12–1.82	—	520/985	52.8%	0.93	0.73–1.18	—
University degree or higher	1317/3172	41.5%	236/562	42.0%	0.86	0.63–1.17	—	140/322	43.5%	1.03	0.70–1.52	—	518/1224	42.3%	1.41	1.10–1.80	—	332/985	33.7%	0.58	0.45–0.74	—
Smoking																						
Never smoker	1264/3213	39.3%	162/566	28.6%	1		< 0.0001	84/324	25.9%	1	0.33–0.59	< 0.0001	291/1240	23.5%	1		< 0.0001	0/1011	0.0%	1		
Ex‐smoker	938/3213	29.2%	177/566	31.3%	1.58	1.25–2.00	—	100/324	30.9%	1.67	1.23–2.27	—	347/1240	28.0%	1.95	1.62–2.35	—	0/1011	0.0%	—	—	—
Current	1011/3213	31.5%	227/566	40.1%	1.97	1.58–2.46	—	140/324	43.2%	2.26	1.70–3.01	—	601/1240	48.5%	4.88	4.01–5.93	—	1011/1011	100.0%	—	—	—
Recreational drugs used^a^																						
None	2016/3258	61.9%	287/568	50.5%	1		0.0017	149/325	45.9%	1		< 0.0001	0/1242	0.0%	—		—	410/1011	40.6%	1		< 0.0001
1 or 2 non‐injection	696/3258	21.4%	163/568	28.7%	1.50	1.21–1.87	—	98/325	30.2%	2.01	1.53–2.63	—	696/1242	56.0%	—	—	—	337/1011	33.3%	3.59	2.98–4.31	—
3+ non‐injection	472/3258	14.5%	102/568	18.0%	1.34	1.04–1.73	—	70/325	21.5%	2.13	1.57–2.89	—	472/1242	38.0%	—	—	—	226/1011	22.4%	3.52	2.84–4.34	—
Injection drug use	74/3258	2.3%	16/568	2.8%	1.46	0.82–2.62	—	8/325	2.5%	1.51	0.71–3.20	—	238/1242	6.0%	—	—	—	74/1011	3.8%	4.02	2.52–6.43	—
Sexual behaviour																						
Condom‐less vaginal or anal sex^a^	1082/3258	33.2%	202/568	35.7%	1.02	0.84–1.24	0.8041	128/325	39.4%	1.32	1.04–1.67	0.0218	625/1242	50.3%	3.46	2.95–4.05	< 0.0001	384/1011	38.0%	1.33	1.14–1.55	0.0004
> 10 new partners in last 12 months	554/3258	17.0%	113/518	21.8%	1.14	0.90–1.45	0.2690	79/305	25.9%	1.61	1.11–1.93	0.0009	413/1173	35.2%	6.43	5.15–8.04	< 0.0001	208/928	22.4%	1.42	1.17–1.73	0.0004
‘Less likely to use a condom’	444/3258	13.6%	93/541	17.2%	1.27	0.96–1.60	0.1040	50/314	15.9%	1.21	0.81–1.55	0.4882	236/1212	19.4%	1.88	1.53–2.31	< 0.0001	138/970	14.2%	0.95	0.77–1.18	0.6657

Quoted *P*‐values refer to those from calculation of χ^2^. Odds Ratios (OR) are unadjusted.

ART, antiretroviral therapy; CI, confidence interval; GAD‐7, General Anxiety Disorder‐7; MSM, men who have sex with men; OR, odds ratio; PHQ‐9, Patient Health Questionnaire‐9.

^a^ In the previous 3 months.

The distribution of responses for questions related to alcohol use are shown in Table 2. Drinking > 20 units alcohol/week was much more prevalent among men than among women, being reported by 12.6% (*n* = 282) of MSM, 8.8% (*n* = 32) of heterosexual men, and only 1.8% (*n* = 11) of women. ‘Alcohol dependence’ (current drinkers with CAGE score ≥ 2) was found in 19.4% (*n* = 435) of MSM, 18.6% (*n* = 68) of heterosexual men, and 10.5% (*n* = 65) of women.

**TABLE 2:** Distribution and interrelationships of alcohol use and dependency variables

Alcohol use	Total		Participants reporting > 20 units alcohol/week from AUDIT questions^a^		Participants reporting alcohol dependence: CAGE score ≥ 2 and current alcohol consumption	
AUDIT: alcohol frequency						
Never	576/3212	17.9%	0/325	0.0%	0/566	0.0%
Monthly or less	681/3212	21.2%	0/325	0.0%	66/566	11.7%
Two toi four times Monthly	690/3212	21.5%	0/325	0.0%	95/566	16.8%
Two to three times Weekly	694/3212	21.6%	38/325	11.7%	161/566	28.4%
Four‐plus times a week	571/3212	17.8%	287/325	88.3%	244/566	43.1%
AUDIT: units when drinking						
Never	576/3160	18.2%	0/325	0.0%	0/561	0.0%
1 or 2	988/3160	31.3%	0/325	0.0%	81/561	14.4%
3 or 4	751/3160	23.7%	0/325	0.0%	137/561	24.4%
5 or 6	464/3160	14.7%	143/325	44.0%	156/561	27.8%
7–9	234/3160	7.4%	77/325	23.7%	105/561	18.7%
10 or more	147/3160	4.7%	105/325	32.3%	82/561	14.6%
AUDIT score						
0	637/3221	19.8%	—		0/561	0.0%
1 or 2	830/3221	25.8%	—		65/561	11.6%
3 or 4	907/3221	28.2%	—		130/561	23.2%
5 or 6	665/3221	20.6%	143/325	44.0%	248/561	44.2%
7 or 8	182/3221	5.7%	182/325	56.0%	118/561	21.0%
AUDIT: 20+ units Weekly	325/3160	10.3%	325/325	100.0%	187/561	32.9%
CAGE: answered ‘yes’ to						
Have you ever felt you should cut down your drinking?	974/3221	30.2%	260/325	80.0%	545/568	96.0%
Have people annoyed you by criticizing your drinking?	295/3221	9.2%	110/325	33.8%	263/568	46.3%
Have you ever felt bad or guilty about your drinking?	530/3221	16.5%	158/325	48.6%	484/568	85.2%
Have you ever had a drink first think in the morning to steady your nerves or get rid of a hangover?	192/3221	6.0%	59/325	18.1%	144/568	25.4%
CAGE score						
0	2103/3221	65.3%	50/325	15.4%	0/568	0.0%
1	547/3221	17.0%	88/325	27.1%	0/568	0.0%
2	332/3221	10.3%	96/325	29.5%	330/568	58.1%
3	176/3221	5.4%	57/325	17.6%	176/568	31.0%
4	63/3221	2.0%	34/325	10.5%	62/568	10.9%
Alcohol dependency CAGE score ≥ 2 and current alcohol consumption	568/3221	17.6%	187/325	57.5%	568/568	100.0%

^a^ Derived from multiplication of alcohol frequency and units when drinking. AUDIT score deriver from modifiedversion of AUDIT‐Cusing first two questions only.

Recreational drugs in the previous 3 months was reported by 38.1% (*n* = 1242) of responders. Use was reported more frequently by MSM (50.6%, *n* = 1138) than by heterosexual men (16.1%, *n* = 60) or women (6.9%, *n* = 44). Injection drug use was uncommon occurring in 3.0% (*n* = 68) of MSM, 1.3% (*n* = 5) of heterosexual men and 0.2% (*n* = 1) of women. Current smoking was also more prevalent among men than women, being reported by 37.1% (*n* = 829) of MSM, 31.6% (*n* = 115) of heterosexual men and 10.9% (*n* = 67) of women.

### Association of factors with alcohol, smoking and recreational drug use

In the univariable analysis, alcohol dependence was associated with being male, younger age, never having started ART, lower levels of social support, symptoms of depression and anxiety, not having money for basic needs, use of recreational drugs and current and ex‐smoking. (Table 1) Similarly, drinking > 20 units/week was associated with being male, being MSM, symptoms of anxiety, current and ex smoking and recreational drug use, but was also associated with older age, white ethnicity, having money for basic needs, condom‐less sex and having more than 10 new partners in the past 12 months.

Recreational drug use in the preceding 3 months was associated in univariable analysis with being male, MSM, younger age, white ethnicity, CD4 count ≥ 500/mL, never having started ART, depression symptoms, having money for basic needs, having formal qualifications, alcohol use and dependency, and current and ex‐smoking, and had strong associations with condom‐less sex, having more than 10 new partners in the past 12 months, and lower condom use with casual partners. (Table 1).

Patterns of association with current smoking were similar to those for the alcohol variables. Current smoking was associated with being male, MSM, of younger age, white ethnicity, CD4 count ≥ 500/mL, never having started ART, having lower social support, symptoms of depression and anxiety, not having money for basic needs, lower education level, alcohol use and dependency, recreational drug use, condom‐less sex, and having more than 10 new partners in the past 12 months.

### Associations of alcohol, smoking and recreational drug use with ART non‐adherence

At the time of recruitment, 76.8% (*n* = 2459) of individuals were on ART having started at least 6 months previously, with a median (IQR) time since initiation of treatment of 7.1 (3.0–12.4) years. Of these, 90.4% (*n* = 2224) had a viral load supressed to below the limit of detection (≤ 50 copies/mL). Of 2418 (98.3%) individuals who responded to questions relating to missing doses, adherence was generally high; 87.8% of participants reported never having missed two or more consecutive days of ART in the past three months, while 12.2% (*n* = 295) reported having done so on two or more occasions and 4.7% (*n* = 113) reported having done so on four or more occasions.

**FIGURE 1:**
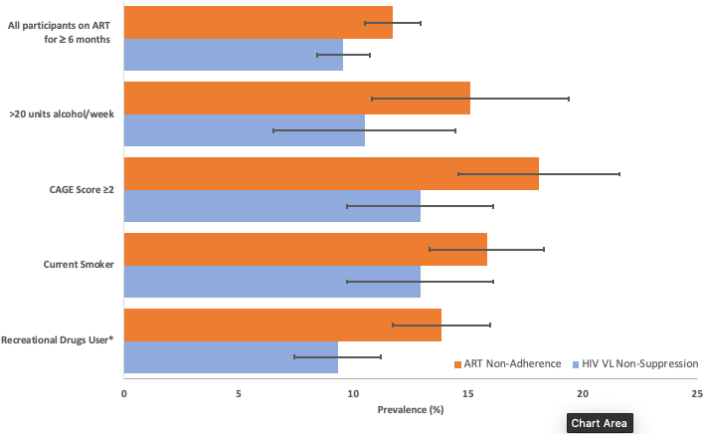
Prevalence of antiretroviral therapy (ART) non‐adherence and viral non‐suppression separated in people on ART who started on ART at least 6 months previously, as well as in those who additionally consumed: > 20 units alcohol/week, had a CAGE score ≥ 2 and were currently drinking alcohol, were current smokers and were recreational drug users. Data are prevalence (%) and 95% confidence intervals. *Use in the last 3 months

We analysed separately the cross‐sectional association of alcohol, smoking and recreational drug use with non‐adherence to ART. Unadjusted associations are shown in Fig. 1 and Table 3. Patterns of association were broadly similar with the two adjustment strategies. Controlling for age, gender/sexual orientation, ethnicity, financial hardship and education, non‐adherence was associated with higher AUDIT alcohol score, drinking > 20 units alcohol, higher CAGE dependency score, alcohol dependence, current smoking, injection and other recreational drug use (Table 3).

**TABLE 3:** Cross‐sectional association of lifestyle factors (alcohol use, recreational drug use, smoking) with antiretroviral therapy (ART) non‐adherence among people on anitretroviral therapy (ART) who started ART at least 6 months prior to the questionnaire. *N* = 2418

	n	Column %	Unajusted Odds Ratio (OR) of non‐adherence			aOR of non‐adherence (age, gender/sexual orientation and ethnicity)			aOR of non‐adherence (age, gender/sexual orientation, ethnicity, hardship and education)		
			OR	95% CI	*P*(LR)	aOR	95% CI	*P* (LR)	aOR	95% CI	*P (LR)*
AUDIT points					*P* = 0.457 Linear test for trend, *P* = 0.621			*P* = 0.013 Linear test for trend, *P* = 0.004			*P* = 0.0074 Linear test for trend, *P* = 0.002
0	473/2413	19.6%	1			1			1		
1 or 2	649/2454	26.9%	0.84	0.58–1.20	0.333	0.91	0.63–1.30	0.588	0.98	0.67–1.42	0.924
3 or 4	696/2454	28.8%	0.79	0.55–1.12	0.187	1.01	0.70–1.47	0.949	1.21	0.82–1.77	0.340
5 or 6	472/2454	19.6%	1.04	0.72–1.51	0.839	1.56	1.05–2.33	0.029	1.80	1.19–2.71	0.005
7–8	123/2454	5.1%	1.20	0.71–2.02	0.884	1.83	1.05–3.19	0.033	1.94	1.10–3.42	0.022
> 20 units alcohol/week											
No	2185/2410	90.7%	1			1			1		
Yes	225/2410	9.3%	1.39	0.97–1.99	0.070	1.77	1.21–2.58	0.005	1.79	1.22–2.63	0.003
Alcohol dependence^a^											
No	1996/2413	82.7%	1			1			1		
Yes	417/2413	17.3%	2.01	1.53–2.64	< 0.001	2.20	1.66–2.92	< 0.001	2.15	1.61–2.88	0.011
CAGE score											
0	a592/2413	66.0%	1		*P* = 0.020 Linear test for trend, *P* < 0.001	1		*P* < 0.001 Linear test for trend, *P* < 0.001	1		*P* < 0.001 Linear test for trend, *P* < 0.001
1	404/2413	16.7%	1.16	0.83–1.61	0.361	1.18	0.84–1.66	0.345	1.11	0.79–1.58	0.274
2	247/2413	10.2%	1.64	1.14–2.38	0.008	1.80	1.24–2.62	0.002	1.75	1.19–2.60	0.220
3	129/2413	5.4%	2.18	1.41–3.39	0.001	2.46	1.56–3.67	< 0.001	2.43	1.53–3.87	0.181
4	42/2413	1.7%	4.66	2.54–7.54	< 0.001	5.10	2.72–9.56	< 0.001	4.20	2.19–8.05	0.003
Smoking status											
Never	964/2406	40.1%	1		*P* < 0.001	1		*P* < 0.001	1		*P* < 0.001
Ex	724/2406	30.1%	0.78	0.57–1.07	0.125	1.12	0.80–1.56	0.519	1.08	0.76–1.52	0.670
Current	718/2406	29.8%	1.56	1.19–2.05	0.001	2.20	1.61–3.01	< 0.001	1.82	1.31–2.52	< 0.001
Recreational drugs used^b^											
None	1520/2418	62.9%	1	*P < 0.001*		1	*P < 0.001*		1	*P < 0.001*	
1 or 2 non‐injection	518/2418	21.4%	1.20	0.90 −1.61	0.213	1.62	1.16–2.27	0.005	1.54	1.10–2.17	0.013
3+ non‐injection	328/2418	13.6%	1.32	0.95–1.85	0.102	1.96	1.32–2.89	0.001	2.03	1.36–3.02	0.001
Injection drug use	52/2418	2.2%	3.97	2.26–6.99	< 0.001	5.71	3.11–10.49	< 0.001	5.48	2.93–10.27	< 0.001

aOR, adjusted odds ratio (in first models adjusted for gender/sexual orientation, ethnicity and age; in second models additionally adjusted for financial hardship and education); CI, confidence interval; LR, likelihood ratio; AUDIT alcohol consumption score derived from modified version of AUDIT‐C using first two questions only

^a^ Alcohol dependence: CAGE score ≥ 2 and current alcohol consumption.

^b^ Drugs use in the previous 3 months. ART non‐adherence was defined as missing ≥ 2 consecutive days of ART on two or more occasions in the past 3 months.

### Associations of alcohol, smoking and recreational drug use with viral non‐suppression

We analysed separately cross‐sectional associations of alcohol, smoking and recreational drug use with viral non‐suppression (VL > 50 copies/mL) in participants who were on ART, having started at least 6 months previously (*n* = 2459). Of these 9.6% (*n* = 235) did not have VL suppression (≤50 copies/mL) at the time of the questionaire. Figure 1 and Table 4 show unadjusted associations. In the unadjusted analysis, we found that viral non‐suppression (VL > 50 copies/mL) was associated with higher CAGE dependency score, alcohol dependence, use of injection recreational drugs and being a current smoker. There was a J‐shaped association with the AUDIT alcohol score, with lower prevalence of VL non‐suppression among those with an intermediate score than among those with a low or high levels of alcohol consumption. In adjusted analysis, additionally controlled for age, gender/sexual orientation, ethnicity, financial hardship and education, most associations remained although the association with AUDIT score was attenuated. Drinking > 20 units of alcohol/week and non‐injection recreational drug use were not associated with viral non‐suppression in any analysis (see Table 4).

There was no evidence of interactions between each of the lifestyle factors and gender/sexual orientation in terms of the association with viral non‐suppression or ART adherence.

**TABLE 4:** Cross‐sectional association of lifestyle factors (alcohol use, recreational drug use, smoking) with viral non‐suppression among people on antiretroviral therapy (ART) who started ART at least 6 months prior to the viral load (VL) measurement. *N* = 2459

*n* = 2459			Unadjusted odds ratio (OR) of detectable VL			aOR of detectable VL (age, gender/sexual orientation and ethnicity)			aOR of detectable VL (age, gender/sexual orientation, ethnicity, hardship and education)		
Risk factor	*n*	Column %	OR	95% CI	*P*(LR)	aOR	95% CI	*P* (LR)	aOR	95% CI	*P* (LR)
AUDIT score											
					*P* = 0.038 Linear test for trend, *P* = 0.919			*P* = 0.081 Linear test for trend, *P* = 0.435			*P* = 0.117 Linear test for trend, *P* = 0.504
0	483/2454	19.7%									
1 or 2	662/2454	27.0%	0.78	0.54–1.14	0.206	0.85	0.57–1.23	0.381	0.94	0.63–1.41	0.773
3 or 4	707/2454	28.8%	0.55	0.37–0.82	0.003	0.65	0.42–0.99	0.041	0.70	0.45–1.09	0.111
5 or 6	477/2454	19.4%	0.61	0.57–1.28	0.455	1.08	0.69–1.67	0.764	1.23	0.78–1.92	0.380
7–8	125/2454	5.1%	0.93	0.50–1.72	0.803	1.16	0.61–2.22	0.641	1.12	0.57–2.22	0.738
> 20 units alcohol/week											
No	2221/2450	90.7%	1						1		
Yes	229/2450	9.3%	1.12	0.72–1.75	0.615	1.32	0.83–2.08	0.246	1.23	0.77–1.96	0.396
Alcohol dependence^a^											
No	2028/2454	82.6%	1						1		
Yes	426/2454	17.4%	1.52	1.10–2.08	0.011	1.54	1.11–2.13	0.011	1.52	1.09–2.13	0.018
CAGE Score											
0	1615/2454	65.8%	1		*P* = 0.022 Linear test for trend, *P* = 0.001	1		*P* = 0.030 Linear test for trend, *P* = 0.002	1		*P* = 0.073 Linear test for trend, *P* = 0.001
1	413/2454	16.8%	0.80	0.53–1.19	0.274	0.80	0.53–1.20	0.282	0.80	0.53–1.20	0.289
2	247/2454	10.1%	1.27	0.82–1.92	0.220	1.30	0.84–2.00	0.173	1.32	0.84–2.04	0.229
3	136/2454	5.5%	1.41	0.82–2.38	0.181	1.43	0.83–2.44	0.166	1.41	0.81–2.44	0.230
4	43/2454	1.8%	2.94	1.45–6.25	0.003	2.78	1.32–5.88	0.017	2.33	1.09–5.00	0.029
Smoking status											
Never	982/2446	40.2%	1		*P*< 0.001	1		*P* = 0.001	1		*P* = 0.005
Ex	731/2446	29.9%	0.74	0.52–1.06	0.097	0.89	0.61–1.30	0.552	0.89	0.60–1.32	0.561
Current	733/2446	30.0%	1.49	1.10–2.03	0.010	1.71	1.21–2.41	0.002	1.58	1.10–2.27	0.013
Recreational drugs used^b^											
None			1		*P*= 0.167	1		*P*= 0.007	1		*P*= 0.263
1 or 2 non‐injection	527/2459	21.9%	0.87	0.61–1.23	0.432	0.96	0.66–1.39	0.836	0.91	0.62–1.35	0.690
3+ non‐injection	332/2459	13.8%	0.93	0.61–1.40	0.711	1.01	0.64–1.60	0.928	1.00	0.62–1.59	0.972
Injection drug use	53/2459	2.2%	2.17	1.07–4.40	0.032	2.25	1.08–4.69	0.031	2.11	1.00–4.47	0.051

aOR, adjusted odds ratio (adjusted for gender/sexual orientation, ethnicity and age); CI, confidence interval; LR, likelihood ratio; AUDIT alcohol consumption score derived from modified version of AUDIT‐C using first two questions only. Detectable VL was defined as VL > 50 copies/mL

^a^ Alcohol dependence: CAGE score ≥ 2 and current alcohol consumption.

^b^ Drugs use in the previous 3 months.

### Associations of alcohol, smoking and recreational drug use with subsequent viral rebound

Of the 899 recruited individuals from the Royal Free clinic, 802 (89.2%) consented to linkage with routine clinic longitudinal data. Prevalence of the lifestyle variables within demographic groups was similar to the overall cohort (see Table 5 footnote).

**TABLE 5:** Longitudinal association of lifestyle factors with hazards of viral rebound (> 200 copies/mL) during follow‐up in people from the Royal Free study site who consented to data linkage, were on ART with VL ≤ 50 copies/mL at study entry and started ART at least 6 months prior to the baseline VL measurement. *N* = 592

*n* = 592			Unadjusted hazards ratio (HR) of viral rebound			aHR of viral rebound (age, gender/sexual orientation and ethnicity)			aHR of viral rebound (age, gender/sexual orientation, ethnicity, hardship and education)		
Risk factor	n	Column %	HR	95% CI	*P* (LR)	aHR	95% CI	*P* (LR)	aHR	95% CI	*P* (LR)
AUDIT points											
			*P* = 0.794 Linear test for trend, *P* = 0.592			*P* = 0.452 Linear test for trend, *P* = 0.051			*P* = 0.332 Linear test for trend, *P* = 0.031		
0	122/591	20.6%	1			1			1		
1 or 2	151/591	25.6%	1.12	0.64–1.96	0.681	1.23	0.70–2.16	0.468	1.38	0.77–2.48	0.283
3 or 4	175/591	29.6%	0.85	0.48–1.49	0.563	1.11	0.61–2–04	0.724	1.18	0.64–2.17	0.590
5 or 6	126/591	21.3%	0.99	0.55–1.81	0.986	1.38	0.73–2.61	0.321	1.33	0.70–2.54	0.376
7–8	17/591	2.9%	1.44	0.49–4.19	0.506	3.02	0.99–9.19	0.052	3.65	1.19–11.19	0.024
> 20 units alcohol/week^a^											
No	542/590	91.9%	1			1			1		
Yes	48/590	8.1%	1.18	0.62–2.27	0.622	1.60	0.82–3.00	0.195	1.40	0.72–2.74	0.345
Alcohol dependence^a^											
No	486/591	82.2%	1			1			1		
Yes	105/591	17.8%	1.44	0.90–2.28	0.126	1.65	1.03–2.63	0.045	1.66	1.03–2.66	0.045
					*P*= 0.210 Linear test for trend, *P*= 0.006			*P*= 0.151 Linear test for trend, *P*= 0.005			*P* = 0.257 Linear test for trend, P = 0.041
CAGE score											
0	399/591	67.5%	1			1			1		
1	87/591	14.7%	0.81	0.44–1.46	0.490	0.89	0.49–1.62	0.701	0.87	0.48–1.58	0.641
2	59/591	10.0%	1.16	0.62–2.20	0.640	1.42	0.75–2.71	0.283	1.63	0.85–3.13	0.145
3	36/591	6.1%	1.31	0.63–2.74	0.459	1.46	0.70–3.06	0.312	1.35	0.64–2.82	0.429
4	10/591	1.7%	3.66	1.33–10.06	0.012	3.86	1.39–10.68	0.009	2.63	0.92–7.51	0.071
Smoking status											
Never	213/588	36.2%	1		*P* = 0.448	1		*P* = 0.251	1		*P* = 0.580
Ex	189/588	32.1%	1.00	0.62–1.62	0.996	1.30	0.79–2.15	0.306	1.30	0.78–2.16	0.310
Current	186/588	31.6%	1.30	0.82–2.06	0.261	1.50	0.92–2.43	0.102	1.20	0.72–1.99	0.490
Recreational drugs used^b^											
None	322/592	54.4%	1		*P* = 0.192	1		*P = 0.007*	1		*P = 0.013*
1 or 2 non‐injection	147/592	24.8%	0.68	0.40–1.16	0.155	0.97	0.54–1.74	0.922	0.82	0.46–1.50	0.538
3+ non‐injection	106/592	17.9%	1.47	0.91–2.35	0.113	2.16	1.22–3.82	0.008	1.82	1.12–3.57	0.020
Injection drug use	17/592	2.9%	2.49	1.07–5.78	0.034	3.37	1.36–8.39	0.009	2.73	1.08–6.89	0.033

aHR, adjusted hazard ratio (in first models adjusted for gender/sexual orientation, ethnicity and age; in second models additionally adjusted for financial hardship and education); CI, confidence interval; LR, likelihood ratio. AUDIT alcohol consumption score derived from modified version of AUDIT‐C using first two questions only.

^a^ Alcohol dependence: CAGE score ≥ 2 and currently drinking.

^b^ Drug use in the 3 months prior to taking the questionnaire. Viral rebound was defined as first VL > 200 copies/mL. In the Royal Free clinic cohort 802 patients consented to prospective data collection. Of these, 596 (74.3%) were MSM, 75 (9.4%) were heterosexual men and 131 (16.3%) were women. Mean (± SD) age was 46.5 (± 9.1) years; 615 (76.7%) were white and 100 (12.5%) were of black African ethnicity. A total of 55 (8.4%) of the cohort drank > 20 units alcohol/week, while 112 (17.2%) were classified as having alcohol dependence; 203 (31.2%) reported being current smokers at the time of questionnaire, and 296 (45.1%) reported taking recreational drugs in the past 3 months; 73.8% (*n* = 592) of individuals with a viral load (VL) < 50 HIV‐1 RNA copies/mL and who had started ART at least 6 months before the VL measurement were included in the above analysis.

Of the 73.8% (*n* = 592) of individuals with a HIV VL ≤ 50 copies/mL and who had started ART at least 6 months before the baseline VL measurement, the median (IQR) follow‐up of participants in the cohort was 7.2 (6.8–7.4) years; 17.7% (*n* = 105) had an episode of viral rebound (> 200 copies/mL) in the observation period (rebound rate = 2.72/100 person‐years). The unadjusted and adjusted Cox‐regression analyses are shown in Table 5. Results were broadly similar with the two adjustment strategies. Using a model adjusted for age, gender/sexual orientation and ethnicity, financial hardship and education, the factors associated with risk of viral rebound were AUDIT > 6, alcohol dependence, higher dependency score, using three or more non‐injection drugs and use of injection drugs (see Table 5).

## DISCUSSION

Our findings demonstrate that PLWH on ART who have evidence of alcohol dependency, inject recreational drugs or currently smoke are at an increased risk of ART non‐adherence and viral non‐suppression, independent of demographic and socioeconomic factors. Longitudinal analysis revealed that among those initially virally suppressed on ART, evidence of alcohol dependency, injection drug use and use of 3 or more non‐injection drugs also predicted risk of viral rebound (> 200 copies/mL).

We found that prevalence of any current alcohol consumption in PLWH was broadly similar to that seen in the general UK population (82.1 *vs*. 79.6%) [24]. There are limited data on alcohol use in PLWH in the UK [25]; however, studies from US [2, 5, 13], Canada [11], and France [12] have also found an association between high alcohol intake and viral non‐suppression. These studies used a similar cut‐off for viral non‐suppression, controlled for other factors influencing adherence and relied on participant‐reported alcohol use. However, cohort sample sizes were smaller than ours with only two studies recruiting > 500 PLWH [2, 12]. Furthermore, heterosexual men and women were under‐represented in previous studies despite marked differences in alcohol use between gender groups, and several did not exclude participants with < 6 months’ ART use [2, 5, 10, 11]. Previous studies also employed different strategies for classifying high alcohol use, some of which were unvalidated [10, 11, 12], hampering meta‐analysis of existing literature [19]. Cultural, financial and social determinants for alcohol consumption vary between nations and influence definitions of alcohol misuse. This underlines the importance of collecting data on large, real‐world samples to inform targeted management strategies.

Despite finding an association of alcohol dependence with viral non‐suppression and viral rebound, this was not the case for consumption of > 20 units alcohol/week, which was only associated with non‐adherence. There may be several reasons for this. First, inaccurate self‐reporting of alcohol use is a significant issue; in the UK it has been estimated that reported intake may only reflect half the true consumption [26]. There is no international consensus on hazardous alcohol consumption – 20 units was chosen in our study as it is 1.5 times the UK recommended alcohol intake for men and women [27], but this might be conservative. In addition, the level of alcohol consumption as characterized by the AUDIT score had a J‐shaped association with viral non‐suppression, with higher prevalence of non‐suppression in non‐drinkers and heavy drinkers than in those with intermediate drinking level; use of the binary classification (drinking >20 units or not) therefore weakened the association. In the longitudinal analysis, there was also evidence that the very highest AUDIT score category of alcohol consumption (score 7–8) was associated with increased risk of viral rebound compared with other levels among those initially suppressed on ART.

The level of smoking was twice that reported contemporaneously for the UK population, although demographic differences confound this comparison [28]. Notably in our study alcohol use and dependency, smoking and drug use were considerably greater in men. A recent systematic review also found that, globally, men living with HIV were more likely to smoke than women; however this trend varied by nation, with sub‐analysis of USA cohorts finding no difference between genders [29]. The association we found between being a current smoker and having a non‐supressed VL has previously been reported in cohorts from the USA [30, 31] and Russia [32].

Poor adherence to ART is critical in viral non‐suppression, and on multivariable analysis we found that alcohol dependence, drinking > 20 units alcohol/week, being a current smoker, and use of injection and other recreational drugs were all associated with ART non‐adherence. Several studies have found that alcohol, recreational drug use and smoking negatively impacts ART adherence in PLWH [3, 16, 18, 19]. In the case of alcohol and drug use this could logically be due to impairment of cognition and/or low mood after drinking or drug‐taking, resulting in an individual forgetting or actively choosing not to take ART. Although we found an association between recreational drug use and ART non‐adherence, we only found evidence of an association with viral non‐suppression in PLWH who injected drugs. Logically non‐injection drug use may also be expected to impair decision‐making. This discrepancy may be due to the pattern or frequency of drug‐taking episodes compared with drinking episodes or other differences between the specific subgroups that use drugs *vs*. alcohol; this should be a focus for future research. The importance of other factors beyond non‐adherence in causing viral non‐suppression remains debatable. It is of interest that we found an association between drinking > 20 units alcohol/week and non‐adherence, but not with viral non‐suppression. It is possible that different ART regimens allowed for a greater degree of non‐adherence in some participants. Alternatively, less frequent ‘binge’ drinking episodes may result in fewer consecutive non‐adherent days. Some groups have suggested that alcohol may have direct effects on viral replication [33]; however, the relevance of these findings in patients on treatment who rarely miss doses is unclear.

The reasons for the association between smoking and poor adherence are less obvious. Significant overlap is seen between smoking and both alcohol misuse and using recreational drugs, which may be pertinent. It is also possible that the increased prevalence of social deprivation and depressive symptoms seen in smokers may have a role. In our study, further adjustment for socioeconomic markers resulted in some attenuation of the association of smoking with non‐adherence compared with results adjusted for core demographic factors only. Reduced medication adherence in smokers has also been reported in diabetes and hypertension [34, 35]. There is also evidence that constituents of cigarette smoke can promote HIV‐1 gene expression in certain individuals [36]. It appears that the effects of smoking on viral suppression are less pronounced than those of alcohol as we found no association between smoking and higher cut‐off for viral rebound of > 200 copies/mL. It remains credible that excess alcohol intake and the consumption of cigarette or recreational drugs are not causal in viral non‐suppression but are more frequent behaviours for a subgroup of PLWH who are less likely to be adherent to ART due to enmeshed psycho‐social factors. Further work is necessary to understand these complex relationships.

Regardless of a causal link, these findings have significant implications, as alcohol, smoking and recreational drug use can all have a deleterious effect on health. Evidence suggests PLWH are at particular risk, with drinking any amount of alcohol being associated with an impact on mortality and physiological health, in excess of that seen for the general population [9]. We have recently shown that smoking, use of injection drugs and level of alcohol consumption (with a J‐shaped association) are associated with an increased hazard of hospital admission of PLWH [37]. Our findings also have potential implications on transmission of HIV/sexually transmitted infections as we also found that those with high alcohol use or who took recreational drugs were more likely to report condom‐less sex and high partner numbers, as well as being more likely to have ART non‐adherence, non‐suppressed VL or VL rebound. The importance of low‐level increases in VL remains incompletely understood, particularly when applied to single transitory ‘blips’ in VL between 40 and 200 copies/ml. However, recent studies have suggested that these may be associated with later virological failure and risk of resistance [38].

An increased awareness of the negative impact of alcohol and smoking on PLWH is needed more generally, highlighted by findings that both PLWH and HIV care providers see alcohol and smoking as a low priority in HIV care [39]. Smoking has been shown to exert a higher morbidity and mortality in PLWH, with an increased risk of progression to lung cancer and chronic obstructive pulmonary disease [17]. Those PLWH who smoke are also less likely to quit smoking than the general population, while a recent review highlights a lack of evidence on the effectiveness of cessation strategies in PLWH populations [39]. Management of alcohol and recreational drug misuse and smoking in PLWH remains significantly under‐investigated, with a recent systematic review finding no large sustained effect of psychological interventions, and is an area in need of further research [40]. Pharmacotherapies have been shown to be efficacious in increasing the odds of smoking cessation in PLWH [41], while methadone maintenance therapy has been shown to be efficacious in both reducing opioid dependence and ART discontinuation [42]. These provide useful strategies which could be incorporated in HIV clinics as part of a comprehensive cessation service. Although we did not ask about specific injection drugs in our study, we did ask about drug type for drug use in general. Chemsex drugs (metamphetamine, GHB/GBL, mephedrone) were much more commonly reported in the small group of injection drug users. Further research on the health effects of injection or chemsex drugs is needed. As the majority of PLWH in the UK obtain healthcare needs from specialist clinics, greater integration of drug and alcohol misuse treatment and smoking cessation services with HIV services is vital.

To our knowledge this is the largest published study in the UK to assess the association of alcohol, smoking and drug use with virological outcomes; however, there were some limitations. Most notably, alcohol, drug use and smoking status was self‐reported at a single time point, resulting in a risk of inaccuracy when generalizing over long periods of time. Although the use of both modified AUDIT and CAGE scoring provides standardised methods for assessing alcohol misuse, they do have limitations. The CAGE scoring system asks participants if they had ‘ever’ experienced each statement, and it is conceivable that some could constitute historical dependence. We attempted to mitigate this by including only cases of alcohol dependence who reported current alcohol use. As we only included the first two questions from the AUDIT‐C score in the questionnaire, we were unable to calculate a complete AUDIT‐C score for each participant. The original questionnaires were completed by PLWH in 2011 and 2012, with a sufficient follow‐up period required to explore the longitudinal relationships. However, societal dynamics and individual lifestyle choices are likely to change with time. In the UK population, while there has been an increasing prevalence of young people who are non‐drinkers and non‐smokers, the prevalence rates in other age groups have remained static [24, 28]. Recreational drug products and patterns of use have also changed, with an increased use of amphetamines and synthetic opioids, as well as a potential increase in use and injection of ‘chemsex’ drugs. This all highlights the importance of the ongoing need for clinicians to assess the alcohol, smoking and recreational drug use in patients. While new, more forgiving ART regimens may have reduced the impact of non‐adherence on viral suppression, all individuals in this study were on daily treatment regimens. In order to identify an adherence pattern which was clinically problematic rather than occasional missed doses, we used a strict definition for non‐adherence (≥ 2 consecutive days of missed ART on two or more occasions in the past 3 months).

## SUMMARY

This study of a large, unselected population of PLWH attending UK outpatient HIV services found that PLWH on ART who have evidence of alcohol dependency, who currently smoke or who have recent injection drug use are at increased risk of non‐suppression of HIV viral load, independent of demographic and socioeconomic factors; evidence of alcohol dependency, use of multiple non‐injection drugs or injection drug use also predicted subsequent viral rebound among those who were initially suppressed. Both smoking and excess alcohol use exert significant negative effects on health, which may be accentuated in PLWH. It is therefore vital that HIV services and clinicians should screen for and address alcohol, recreational drug use and smoking. More research is needed to investigate optimal cessation strategies, to explore the association of level of alcohol consumption with HIV outcomes and to assess what amount of alcohol use is safe in PLWH.

## Data Availability

Data are available on request from the authors.
